# Peptide Fraction pOh2 Exerts Antiadipogenic Activity through Inhibition of C/EBP-*α* and PPAR-*γ* Expression in 3T3-L1 Adipocytes

**DOI:** 10.1155/2017/4826595

**Published:** 2017-03-23

**Authors:** Thi Tuyet Nhung Nguyen, Thi Thu Ha, Thi Hoa Nguyen, Thi Hien Vu, Nam Hai Truong, Hoang Ha Chu, Dong Van Quyen

**Affiliations:** Institute of Biotechnology (IBT), Vietnam Academy of Science and Technology (VAST), 18 Hoang Quoc Viet Street, Cau Giay District, Hanoi 100000, Vietnam

## Abstract

Many studies have comprehensively examined the venom of* Ophiophagus hannah* snake. Its venom comprises different compounds exhibiting a wide range of pharmacological activities. In this investigation, four peptide fractions (PFs), ranging from 3 kDa to 10 kDa, isolated from the Vietnamese snake venom of* O. hannah* were separated by HPLC and investigated for their inhibitory activity on adipogenesis in 3T3-L1 adipocytes. The most effective PF was then further purified, generating two peptides, pOh1 and pOh2. Upon investigation of these two peptides on 3T3-L1 adipocytes, it was revealed that, at 10 *μ*g/mL, pOh2 was able to inhibit the lipid accumulation in 3T3-L1 adipocytes by up to 56%, without affecting cell viability. Furthermore, the pOh2 downregulated the gene expression of important transcription factors C/EBP-*α* and PPAR-*γ*. In addition, aP2 and GPDH adipocyte-specific markers were also significantly reduced compared to untreated differentiated cells. Taken together, pOh2 inhibited the expression of key transcription factors C/EBP-*α* and PPAR-*γ* and their target genes, aP2 and GPDH, thereby blocking the adipocyte differentiation. In conclusion, this novel class of peptide might have potential for in vivo antiobesity effects.

## 1. Introduction

Snakes have a long evolutionary history, and their venoms have evolved to allow them to incapacitate, paralyze, and kill their prey. These venomous toxins can interact with receptors/ligands with a high degree of specificity and regulate various physiological processes, making them an excellent source for novel drug leads and designs [1].

Among the venomous snakes,* Ophiophagus* is a monotypic genus with only one species recognized,* Ophiophagus hannah*. However, its vast geographical distribution accompanied by morphological differences suggests potential taxonomic divergence and variation in venom composition. Data of venom gland transcriptome of Malaysian* O. hannah* represent 23 protein families related to venom toxic functions, which were dominated by three finger toxins (3FTxs) (84.9%), followed by snake venom metalloproteases (SVMPs, 3.7%), phospholipases A_2_ (PLA_2_s, 2.2%), cysteine-rich secretory proteins (CRISPs, 2.1%), Kunitz-type proteinase inhibitors (KUNs, 1.8%), L-amino acid oxidases (LAAOs, 1.5%), and many others that are expressed in less than 1% of abundance [2] in which hyaluronidase, DPP-IV, and 5′-nucleotidase were not previously reported in the venom gland transcriptome of a Balinese* O. hannah* [3].

Various compounds from the venom of* O. hannah* have previously been isolated and characterized, including neurotoxins [4], L-amino acid oxidases [5], metalloproteinases [6], 3FTxs [7], phospholipases A2 (PLA2s) [8, 9], ohanin [10], Kunitz-type protease inhibitors [11], and factor X activator [12]. These compounds, exerting remarkable biological properties, are usually secreted with an aim to reach specific targets including receptors, membranes, and enzymes within the inflicted organism. Therefore, these toxins make excellent probes for novel pharmacological tools or therapeutic drugs.

For decades, obesity has been rising at alarming rate, contributing to almost $147 billion to $210 billion dollars in preventable healthcare [13, 14]. In 2016, more than 1.9 billion adults and 41 million children under the age of 5 were overweight or obese. Obesity results from an imbalance between energy intake and expenditure. Obesity is a significant risk factor for and contributor to increased morbidity and mortality, most importantly from cardiovascular disease (CVD) and diabetes and also from cancer and chronic diseases, including osteoarthritis, liver and kidney disease, sleep apnea, and depression [15–17]. Commonly prescribed weight loss medications include Lorcaserin, Orlistat, Phentermine, Topiramate, Buproprion, Naltrexone, and Liraglutide. However, these have short-term benefits for weight loss and are often associated with various side effects (insomnia, increased blood pressure, fast heart rate, oily spotting, headache, dry mouth, and dizziness) and have potential for drug abuse [18]. Rebound weight gain is also considered a major side effect after the cessation of medication use [19]. Therefore, new therapies are always needed. Recently, some alternative therapies related to obesity treatment have drawn attention thanks to their potentials on animals without causing significant side effects. ShK-186 is an analog of a 35-residue peptide ShK, isolated from sea anemone* Stichodactyla helianthus*. ShK-186 selectively blocks the Kv1.3 channel that has been implicated in the regulation of energy homeostasis and body weight [20]. Snake venom also contains lots of potassium channels blockers [21–23]. ShK-186 significantly reduced weight gain, fat accumulation, and associated inflammation in diet-induced obese mice [24]. Bee venom (BV) has been widely used in the treatment of chronic inflammatory diseases. In vitro, BV inhibited lipid accumulation without cytotoxicity in 3T3-L1 cells by downregulation of expression of transcription factors, CCAAT/enhancer-binding proteins (C/EBPs), and peroxisome proliferator-activated receptor-gamma (PPAR-*γ*). BV has also reduced body weight gain in diet-induced obese C57BL/6 mice [25].

In addition, PLA2, first isolated from snake venom, has been demonstrated as an important enzyme in adipose tissue. In the body, PLA2 hydrolyzes fatty acids (FA) from the sn-2 position of phospholipids to generate FA and lysophospholipids [26]. These products are tightly linked to obesity. Therefore, PLA2 may prove useful for potential antiobesity therapies. Until now, hundreds of snake venom PLA2 enzymes have been purified and characterized [27–29]. For these reasons, we may be able to find some agent in the venom of* O. hannah* which could have a potential use in obesity treatment.

Although multiple molecular processes are involved in increasing adipose tissue mass, obesity can be a result of both an increase in adipocyte size as a consequence of increased triacylglycerol content within the fat cell and an increased number of adipocytes resulting from differentiation of precursor cells [30]. For the past 20 years, in vitro systems have been extensively used to study adipocyte differentiation. The most well-characterized and reliable model for studying the conversion of preadipocytes into adipocytes is 3T3-L1 [31]. The formation and appearance of developing fat droplets also mimic live adipose tissue. At confluence, 3T3-L1 preadipocytes express very early markers of adipocyte differentiation. In culture, differentiated 3T3-L1 preadipocytes possess most of the ultrastructural characteristics of adipocytes from animal tissue [31]. Furthermore, at least two families of transcription factors, C/EBPs and PPARs, are induced early during adipocyte differentiation. PPAR-*γ* is largely adipocyte-specific and is expressed at low but detectable levels in preadipocytes. Its expression rapidly increases after hormonal induction of differentiation and is easily detectable during the second day of 3T3-L1 adipocyte differentiation. Understanding how the various molecules regulate adiposity may lead to the development of novel therapeutic approaches to human obesity [32].

In this respect, we screened the venom of Vietnamese snake* O. hannah* with an adipogenesis assay to identify new compounds that could have a therapeutic value in the prevention or treatment of obesity. Using HPLC, four PFs (1, 2, 3, and 4), ranging from 3 kDa to 10 kDa, were fractionated and investigated for their inhibitory activity on adipogenesis in 3T3-L1 adipocytes. The most effective PF2 was then further purified, generating two peptides, pOh1 and pOh2. At 10 *μ*g/mL, pOh2 was able to inhibit the lipid accumulation in 3T3-L1 adipocytes without affecting viability of 3T3-L1 adipocytes. At the genomic level, pOh2 downregulated gene expression of important transcriptional factors C/EBP-*α* and PPAR-*γ*. In addition, expressions of target genes of C/EBP-*α* and PPAR-*γ*, aP2 and GPDH, were significantly reduced in the presence of pOh2.

## 2. Materials and Methods

### 2.1. Fractionation of Peptides

Five grams of lyophilized* O. hannah* (Vietnamese king cobra) crude venom was purchased from Dong Tam Snake Farm (Tien Giang, Ho Chi Minh, Vietnam) and dissolved in PBS and centrifuged, and a clear protein solution was collected. Molecules from the reconstituted venom solution were separated according to their molecular weight by sequential centrifugation using a Centriplus centrifugal filter device with different molecular weight cut-offs (100, 50, 30, 10, and 3 kDa; Millipore). The PFs corresponding to peptides with a molecular mass between 3 and 10 kDa were purified by using semipreparative reversed-phase HPLC with a Vydac C_18_ column (250 × 10 mm; 10 *μ*m; 300 Å). Peptide fraction 2, which showed major adipogenesis-inhibiting activity, was further purified by analytical HPLC. Absorbance was monitored at 220 nm [33].

### 2.2. Cell Culture

3T3-L1 preadipocytes were grown in Dulbecco's Modified Eagle's Medium (DMEM) containing 10% fetal bovine serum (FBS), 1% penicillin (10.000 U/mL), and 1% streptomycin and incubated at 37°C supplemented with 5% CO_2_. Cells were subcultured every two to three days at approximately 80% confluence.

### 2.3. Cell Viability Assay

Cell viability was assessed by MTS assay. The 3T3-L1 preadipocytes were seeded (5000 cells/well) in 96-well plates, grown until confluence, induced to differentiate, and grown to maturation. Cells were then incubated in serum-free medium with different concentrations of peptide fractions for 24 hours. Before assay for cell viability, cells were washed twice with DMEM/10% FBS, and 100 *μ*L of DMEM/10% FBS medium was added to each well with 20 *μ*L of 3-(4,5-dimethylthiazol-2-yl)-5-(3-carboxymethoxyphenyl)-2-(4-sulfophenyl)-2H-tetrazolium salt (MTS) solution per well (CellTiter 96 AQueous One Solution). Cells were incubated for one hour at 37°C; then 25 *μ*L of 10% sodium dodecyl sulfate was added to each well. The absorbance was measured at 490 nm in a plate reader in order to determine the optical density related to the conversion of MTS in the purple water-soluble Formazan, which is proportional to the number of live cells. Results were expressed as a percentage of the control (untreated 3T3-L1 cells) [34].

### 2.4. Adipocyte Differentiation

3T3-L1 preadipocytes were seeded onto 12-well plates at a density of 2 × 10^4^ cells/well. Two days after confluence (defined as day 0), cells were stimulated to differentiate with differentiation DMEM medium containing 10% FBS, 1.0 *μ*M dexamethasone (DXM), 0.5 mM 3-isobutyl-1-methylxanthine (IBMX), and 1.0 *μ*g/mL insulin for two days. At day two, differentiation DMEM medium was replaced with adipogenesis DMEM medium containing 10% FBS and 1 *μ*g/mL insulin and incubated for another two days (day four). Thereafter, the cells were maintained in DMEM medium containing 10% FBS for an additional four days (day eight) with medium changing every two days. In the course of screening adipocyte differentiation-inhibitory activity, the peptide fraction was added two days after confluence (day 0) and maintained during cell differentiation until the time when the cells were harvested for the following described tests [34]. Results were obtained from three independent experiments performed in triplicate.

### 2.5. Oil Red O Staining of 3T3-L1 Adipocyte

Eight days after the differentiation induction, in order to measure the lipid accumulation within the 3T3-L1 adipocytes, the cells were fixed using 10% formaldehyde and stained with Oil Red O. In brief, the cells were washed twice with PBS and stained with 1 mL of Oil Red O solution per well for 15 minutes at room temperature. The cells were then washed twice with 1 mL of wash solution and stained plates were visualized under a microscope. After that, 0.5 mL of dye extraction solution was added and followed by orbital shaking for 15–30 minutes. The lipid accumulation was quantified by eluting stain in the dye extraction solution, followed by spectrophotometric measurement at 510 nm. The percentage of Oil Red O stained material relative to control wells containing cell culture medium without PF was calculated as *A*_510 nm_ (PF treated)/*A*_510 nm_ (control) × 100 [34].

### 2.6. Quantitative Real-Time PCR Analysis

Total RNA was extracted from 3T3-L1 adipocytes (8 days after the induction) using TRIzol reagent (Invitrogen, Life Technologies; Carlsbad, CA, USA) and the concentration of RNA was measured by NanoDrop (Thermo Fisher). The extracted RNA was reverse-transcribed into complementary DNA using a high capacity cDNA reverse transcription kit (Applied Biosystems, Foster City, CA, USA) according to the manufacturer's instructions. Then the RNA expression level was quantified by a quantitative real-time PCR using SYBR Green PCR Master Mix (Applied Biosystems, Woolston, Warrington, UK) and the 7500 real-time PCR system (Applied Biosystems, Foster City, CA, USA) according to the manufacturer's protocol. The sequences of primers used for quantitative real-time PCR were as follows:

CCAAT/enhancer-binding protein-*α* (C/EBP-*α*):

  F: 5′-AGCAACGAGTACCGGGTACG-3′ and
  R: 5′-TGTTTGGCTTTATCTCGGCTC-3′;

Peroxisome proliferator-activated receptor-*γ* (PPAR-*γ*):

  F: 5′-CAAGAATACCAAAGTGCG-ATCAA-3′ and
  R: 5′-GAGCTGGGTCTTTTCAGAAT-AATAAG-3′;

Fatty acid binding protein (aP2):

  F: 5′-AGTGAAAACTTCGATGATTACATGAA-3′ and
  R: 5′-GCCTGCCACTTTCCTTGTG-3′;

Glycerol-3-phosphate dehydrogenase (GPDH):

  F: 5′-CTCT-TCTTGCCGCTTCAGTTT-3′ and
  R: 5′-CATGTAGGCCA-TGAGGTCCACCAC-3′;

Glyceraldehyde 3-phosphate dehydrogenase (GAPDH):

  F: 5′-TGCACCACCAACTGCTTAGC-3′ and
  R: 5′-GGCATGGACTGTGGTCATGAG-3′.

Relative quantification of gene expression with real-time PCR data was calculated in relation to GAPDH [34].

## 3. Results

**Figure 1:**
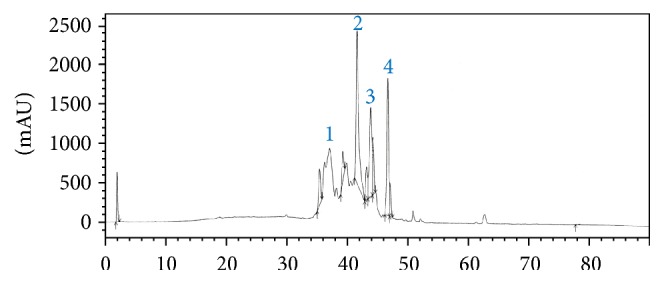
Reversed-phase HPLC of the crude venom of* O. hannah*. The fraction corresponding to peptides with a molecular mass between 3 and 10 kDa was applied to a semipreparative Vydac C_18_ column. 1, 2, 3, and 4 correspond to fractions eluted at 36–39 (fraction 1), 40–43 (fraction 2), 44–46 (fraction 3), and 47–49 (fraction 4) minutes, respectively.

The present study was carried out with the purpose of isolating and characterizing potent adipogenesis-inhibiting peptides from the crude venom of the Vietnamese snake* O. hannah*. Following various purification processes, four main PFs with molecular weights ranging between 3 and 10 kDa were collected from the initial crude snake venom (Figure 1). These fractions, mostly nonhomogenous as determined by analytical RP-HPLC, were screened for their propensity to inhibit adipogenesis in 3T3-L1 cell line.

### 3.1. Effect of PFs on Adipogenesis in 3T3-L1 Adipocytes

**Figure 2:**
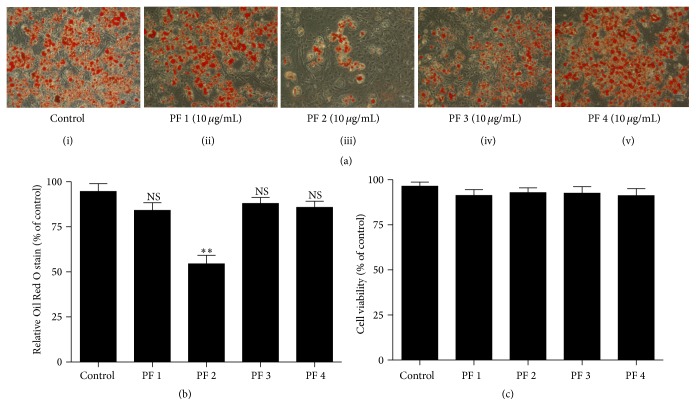
Effect of PFs on adipogenesis in 3T3-L1 adipocytes. (a) Intracellular lipid was stained with Oil Red O on differentiated 3T3-L1 adipocytes treated with adipogenesis cocktail in the presence or absence of PFs (1, 2, 3, and 4) at 10 *μ*g/mL for 8 days (from day 0 to day 8). (b) Quantitative analysis of Oil Red O staining. To determine the extent of adipose conversion, 1 mL Oil Red O solution was added to the 12-well plates. The extracted dye was removed after 15 minutes of incubation at RT and its optical density was monitored at 510 nm. (c) The viability of 3T3-L1 adipocytes. Results are the mean ± SD of three independent experiments conducted in triplicate. NS, not significant; ^*∗∗*^*p* < 0.01 as compared with differentiated 3T3-L1 adipocytes.

To study the effects of each PF on adipogenesis, 3T3-L1 adipocytes, the widely used culture model, were employed [31].  Figure 2(a) (panel (i)) highlighted positive control of 3T3-L1 adipocytes after treatment with an adipogenesis cocktail containing IBMX, dexamethasone, and insulin for a period of eight days, where lipid accumulation was observed inside the 3T3-L1 adipocytes.

As we can see in Figure 2(a) (panel (iii)), at 10 *μ*g/mL, PF2 reduced number of 3T3-L1 cells that accumulated fat droplets, while PF1, PF3, and PF4 (panels (ii), (iv), and (v), resp.) did not show any significant difference compared to the control. The lipid accumulation was significantly reduced by about 45% in the presence of PF2, and this observation was further supported by the quantitative analysis of neutral lipid content as shown in Figure 2(b). Investigation of the effect of four PFs on the viability of 3T3-L1 cells showed that, at a dose of 10 *μ*g/mL, none of the PFs affected the viability of 3T3-L1 adipocytes (Figure 2(c)).

**Figure 3:**
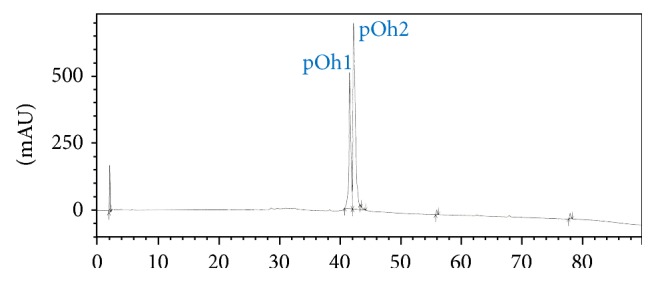
Analytic HPLC purification of the peptide fraction 2 of* O. hannah*. The fraction was applied on an analytical Vydac C_18_ column. 1 and 2 correspond to peaks eluted at 40–42 and 42–44 minutes, respectively.

The active PF2 was therefore selected for further investigations. This PF2 was purified by analytic HPLC. As shown in Figure 3, two peaks, pOh1 and pOh2, were collected (Figure 3).

These two peaks were examined for their effect on adipogenesis in 3T3-L1 cells.

**Figure 4:**
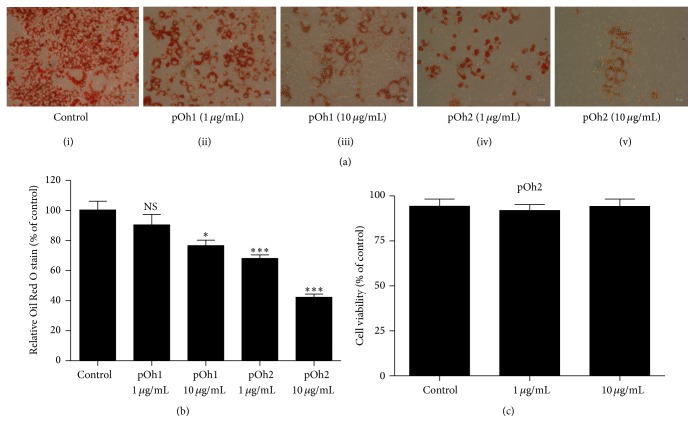
Effect of pOh1 and pOh2 on adipogenesis in 3T3-L1 adipocytes. (a) Intracellular lipid was stained with Oil Red O on differentiated 3T3-L1 adipocytes incubated only with an adipogenesis cocktail (i) or with an adipogenesis cocktail and supplemented with 1 *μ*g/mL of pOh1 (ii), 10 *μ*g/mL of pOh1 (iii), 1 *μ*g/mL of pOh2 (iv), and 10 *μ*g/mL of pOh2 (v). (b) Optical density of extracted dye was monitored at 510 nm. (c) The viability of 3T3-L1 adipocytes. Results shown are the means of three independent experiments conducted in triplicate. NS, not significant; ^*∗∗∗*^*p* < 0.001 and ^*∗*^*p* < 0.05 as compared with differentiated 3T3-L1 adipocytes.

As we can see in Figure 4(a), at two different doses, both pOh1 and pOh2 reduced the number of cells accumulating fat droplets. However, a greater effect was observed with pOh2.

pOh2 significantly reduced intracellular lipid accumulation compared to pOh1 (Figure 4(a)). The measurement of the absorbance at 510 nm of neutral lipid content shows almost 56% reduction when incubated with pOh2 at 10 *μ*g/mL compared to controls, whereas only 20% reduction was observed with pOh1 (Figure 4(b)). No effect on cell viability of 3T3-L1 adipocytes by pOh2 was observed (Figure 4(c)).

### 3.2. The Effect of pOh2 on mRNA Expression of Lipid Metabolism-Related Genes in Differentiated 3T3-L1 Adipocytes

Adipogenesis is a highly regulated process requiring coordinated expression and activation of key transcription factors which include C/EBP-*α* and PPAR-*γ* [32].

**Figure 5:**
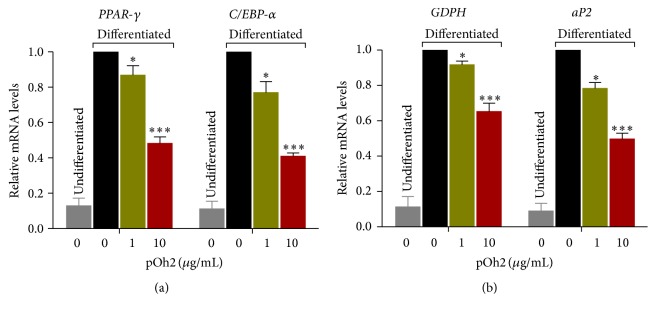
The effect of pOh2 on mRNA expression levels of PPAR-*γ* and C/EBP-*α* (a) and GPDH and aP2 (b) in 3T3-L1 adipocytes. Results shown are the means of three independent experiments conducted in triplicate. NS, not significant; ^*∗∗∗*^*p* < 0.001 and ^*∗*^*p* < 0.05 as compared with differentiated 3T3-L1 cells. PPAR: peroxisome proliferator-activated receptor; C/EBP: CCAAT/enhancer-binding protein; GPDH: glycerol-3-phosphate dehydrogenase; aP2: fatty acid binding protein.

The events depicted in Figure 5(a) revealed that pOh2 treatment significantly reduced the mRNA level of C/EBP-*α* and PPAR-*γ* in differentiated 3T3-L1 adipocytes after eight days of treatment as compared to the control.

aP2 transcription is known to be mediated by a number of transcription factors such as PPAR-*γ* and C/EBP-*α*. The expression levels of PPAR-*γ* and C/EBP-*α* were downregulated at the mRNA level in response to pOh2 treatment. Therefore, we proceeded to investigate whether pOh2 affects aP2 expression. Cells treated with 10 *μ*g/mL pOh2 resulted in a significant decrease in the mRNA levels of aP2 (Figure 5(b)). Moreover, mRNA levels of GPDH, a key regulatory enzyme involved in triglyceride synthesis, were also significantly decreased in pOh2-treated adipocytes compared to those of the positive control cells (Figure 5(b)).

Taken together, pOh2 inhibited the expression of key transcription factors C/EBP-*α* and PPAR-*γ* and their target genes aP2 and GPDH, thereby blocking adipocyte differentiation.

## 4. Discussion

Obesity has become a major health problem worldwide. It is associated with heart disease, hypertension, cancer, and type 2 diabetes mellitus [35]. Current medication for the treatment of obesity has short-term benefits for weight loss via the suppression of appetite, although it is often associated with side effects. That is why alternative therapies for obesity are always necessary.

Venomous animals have a long evolutionary history, and their venoms have a potential for tremendous contribution to the treatment of human diseases [36–38]. However, venom-based medical treatments have only recently received attention [39]. The current study indicated that pOh2, isolated from the venom of Vietnamese* O. hannah* snake, may be a potential candidate for obesity treatment.

Adipose tissue is vitally important to various normal processes of the human body. It is the most prevalent tissue in the human body. Adipose tissue is divided into two subtypes, white fat and brown fat. White fat is widely distributed and it represents the primary site of fat metabolism and storage, whereas brown fat is relatively scarce and its main role is to provide body heat, which is essential for newborn babies. The growth of white adipose tissue is a result of both increased adipocyte size and an increase in adipocyte number, which is called adipocyte differentiation. Adipose tissue also plays an important role in numerous processes through its secretory products and endocrine functions. Although adipose tissue is vitally important to various normal processes of the human body, it has also many implications for human disease states. Obesity is a common health problem in industrialized countries and is considered a major risk factor for non-insulin-dependent diabetes mellitus, cardiovascular diseases, and hypertension. Obesity is an enlargement of adipose tissue to store excess energy intake. Hyperplasia (cell number increase) and hypertrophy (cell size increase) are two possible growth mechanisms [40].

pOh2 at 10 *μ*g/mL significantly decreased the lipid accumulation in 3T3-L1 adipocytes. Using the Oil Red O staining technique, our results showed that pOh2 reduced up to 56% of lipid accumulation compared to the positive control, suggesting that pOh2 possesses obesity inhibitory potential. In line with our observations, several previous studies looking at the adipogenesis also observed that the lipid accumulation was reduced from two to four times between treated and control cell lines [25, 34, 41] and untreated and treated obese mice [42]. Bee venom is an example. Although bee venom has been widely used in the treatment of some immune-related diseases [43], recent studies demonstrated its medicinal utility against obesity. Cheon and coworkers determined that BV inhibited lipid accumulation in 3T3-L1 adipocytes using the Oil Red O staining technique. This venom had no cytotoxic activity towards 3T3-L1 cells at tested doses. Moreover, bee venom administration, within the dose of 0.1 mg/kg to 1 mg/kg, has reduced body weight gain in diet-induced obese C57BL/6 mice after four weeks of treatment [25].

Potassium channels Kv1.3 have been implicated in the regulation of energy homeostasis and body weight. ShK-186 is an analog of ShK, a peptide isolated from sea anemone* Stichodactyla helianthus*. This ShK-186 selectively blocks the Kv1.3 channel. Diet-induced obese C57BL6/J mice were treated with ShK-186 via subcutaneous injection at the dose of 500 *μ*g/kg every other day. After 45 days, in comparison with untreated mice, ShK-186-treated mice exhibited a significant reduction of weight gain, adiposity, and associated inflammation. Whole-body CT scans showed that ShK-186 treatment decreased lipid accumulation, thus inhibiting obesity in mouse model. The authors observed that the lipid-laden vesicles were significantly reduced in averaged size up to four times compared to untreated mice. These studies strongly suggested that there is a significant link between reduction of lipid accumulation and inhibition of obesity. By blocking inflammation, the peptide can combat the harmful effects of obesity. In addition, the treated mice exhibited lower blood levels of cholesterol, glucose, insulin, and leptin and improved glucose tolerance and peripheral insulin sensitivity [24].

Besides, other alternative therapies for obesity such as herbal medicine treatments have long been used in eastern cultures, but few herbal medicines have been investigated to prove their efficacy and mechanisms compared to western drugs [44].

*Achyranthes bidentata* Blume root water extract was shown to have antiadipogenic effects using the same methods as those in this study. Postconfluent 3T3-L1 preadipocytes treated every two days with 10 *μ*g/mL of* Achyranthes bidentata* Blume root water extract for eight days resulted in a 36% reduction of lipid accumulation. Therefore,* Achyranthes bidentata* Blume root water extract was subjected to further analysis to investigate its possible antiadipogenic effects on adipocyte differentiation [40]. In another study, ursolic acid, a phytochemical found in a wide variety of plants and mostly well-known as a comprising part of apple peels, was also shown to inhibit adipogenesis in 3T3-L1 adipocytes in a dose-dependent manner [41]. Following Oil Red O staining in differentiated 3T3- L1 adipocytes, ursolic acid at a dose of 10 *μ*M significantly decreased the lipid content in 3T3-L1 adipocytes by 30% compared to the control. These above studies showed that a reduction of lipid accumulation by up to 30% significantly correlated with antiobesity effect of these compounds. These are strong indicators to imply the potency of pOh2 as an antiobesity agent.

Much focus has been given to the molecular mechanisms to regulate the increase of adipose tissue mass. At a genomic level, the global endemic of obesity is correlated with transcriptional factors such as PPAR-*γ* and C/EBP-*α* which play a significant role in the expression of adipocyte genes and in turn controlling the process of adipocyte differentiation [32]. Therefore, in this regard, pOh2 is investigated to determine its role against the regulation of PPAR-*γ* and C/EBP-*α* genes. It is worth noting that PPAR-*γ* and C/EBP-*α* levels were downregulated in 3T3-L1 adipocytes treated with 10 *μ*g/mL of pOh2. In another study on bee venom, the authors indicated that BV also inhibited adipogenesis by downregulation of transcription factors C/EBP-*α* and PPAR-*γ* [25].

In addition, during adipocyte differentiation, PPAR-*γ* and/or C/EBP-*α* is implicated in the coordinated activation of several adipocyte genes such as aP2 (an adipocyte-specific fatty acid binding protein), GLUT4, and SCD1. pOh2 treatment at 10 *μ*g/mL caused downregulation of aP2 mRNA expression, as well as GPDH, a key regulatory enzyme involved in TG synthesis. Thus, our results suggest that the inhibition of adipogenic gene expression induced by pOh2 may be mediated via the inhibition of PPAR-*γ* and C/EBP-*α* expression.

Concisely, the comprehensive examination of the peptide component of the* O. hannah* venom belongs to a diverse range of protein families with a wider array of functionality [11, 45–47]. Upon examination, results of the study revealed that pOh2 was the most potent in inhibiting the process of adipogenesis by limiting lipid accumulation in the 3T3-L1 adipocytes. This is achieved by downregulating of transcriptional factors PPAR-*γ* and C/EBP-*α* and their downstream target genes, aP2 and GPDH.

## 5. Conclusion

Reptile venoms and toxins have a potential for tremendous contribution to the treatment of human diseases. Each year, several new natural toxins with highly specific actions are discovered. pOh2, isolated from the venom of the Vietnamese snake* O. hannah*, was the first peptide fraction that has been found to act as an inhibiting agent of adipogenesis in 3T3-L1 adipocytes. Further studies are ongoing to investigate the amino acid sequence and structure of pOh2. pOh2 is a promising agent for further research in searching for alternative therapies for the prevention or treatment of obesity.

## References

[1] Berger B. J., Bhatti A. R. (1989). Snake venom components and their cross-reactivity: a review. *Biochemistry and Cell Biology*.

[2] Tan C. H., Tan K. Y., Fung S. Y., Tan N. H. (2015). Venom-gland transcriptome and venom proteome of the Malaysian king cobra (*Ophiophagus hannah*). *BMC Genomics*.

[3] Vonk F. J., Casewell N. R., Henkel C. V. (2013). The king cobra genome reveals dynamic gene evolution and adaptation in the snake venom system. *Proceedings of the National Academy of Sciences of the United States of America*.

[4] He Y.-Y., Lee W.-H., Zhang Y. (2004). Cloning and purification of *α*-neurotoxins from king cobra (*Ophiophagus hannah*). *Toxicon*.

[5] Lee M. L., Fung S. Y., Chung I., Pailoor J., Cheah S. H., Tan N. H. (2014). King cobra (*Ophiophagus hannah*) venom L-amino acid oxidase induces apoptosis in PC-3 cells and suppresses PC-3 solid tumor growth in a tumor xenograft mouse model. *International Journal of Medical Sciences*.

[6] Guo X.-X., Zeng L., Lee W.-H., Zhang Y., Jin Y. (2007). Isolation and cloning of a metalloproteinase from king cobra snake venom. *Toxicon*.

[7] Li J., Zhang H., Liu J., Xu K. (2006). Novel genes encoding six kinds of three-finger toxins in *Ophiophagus hannah* (king cobra) and function characterization of two recombinant long-chain neurotoxins. *Biochemical Journal*.

[8] Xu S., Gu L., Wang Q., Shu Y., Song S., Lin Z. (2003). Structure of a king cobra phospholipase A2 determined from a hemihedrally twinned crystal. *Acta Crystallographica Section D: Biological Crystallography*.

[9] Xu S., Gu L., Wang Q., Shu Y., Lin Z. (2002). Preliminary crystallographic study of an acidic phospholipase A2 from *Ophiophagus hannah* (king cobra). *Acta Crystallographica Section D: Biological Crystallography*.

[10] Pung Y. F., Wong P. T. H., Kumar P. P., Hodgson W. C., Kini R. M. (2005). Ohanin, a novel protein from king cobra venom, induces hypolocomotion and hyperalgesia in mice. *The Journal of Biological Chemistry*.

[11] He Y.-Y., Liu S.-B., Lee W.-H., Qian J.-Q., Zhang Y. (2008). Isolation, expression and characterization of a novel dual serine protease inhibitor, OH-TCI, from king cobra venom. *Peptides*.

[12] Lee W.-H., Zhang Y., Wang W.-Y., Xiong Y.-L., Gao R. (1995). Isolation and properties of a blood coagulation factor X activator from the venom of king cobra (*Ophiophagus hannah*). *Toxicon*.

[13] Cawley J., Meyerhoefer C. (2012). The medical care costs of obesity: an instrumental variables approach. *Journal of Health Economics*.

[14] Finkelstein E. A., Trogdon J. G., Cohen J. W., Dietz W. (2009). Annual medical spending attributable to obesity. *Health Affairs*.

[15] Bhurosy T., Jeewon R. (2014). Overweight and obesity epidemic in developing countries: a problem with diet, physical activity, or socioeconomic status?. *The Scientific World Journal*.

[16] Marks J. B. (2004). Obesity in America: it's getting worse. *Clinical Diabetes*.

[17] Gallus S., Lugo A., Murisic B., Bosetti C., Boffetta P., La Vecchia C. (2015). Overweight and obesity in 16 European countries. *European Journal of Nutrition*.

[18] Kang J. G., Park C.-Y. (2012). Anti-obesity drugs: a review about their effects and safety. *Diabetes and Metabolism Journal*.

[19] Abdollahi M., Afshar-Imani B. (2003). A review on obesity and weight loss measures. *Middle East Pharmacy*.

[20] Beeton C., Pennington M. W., Norton R. S. (2011). Analogs of the sea anemone potassium channel blocker shk for the treatment of autoimmune diseases. *Inflammation and Allergy—Drug Targets*.

[21] De Weille J. R., Schweitz H., Maes P., Tartar A., Lazdunski M. (1991). Calciseptine, a peptide isolated from black mamba venom, is a specific blocker of the L-type calcium channel. *Proceedings of the National Academy of Sciences of the United States of America*.

[22] Possani L. D., Martin B. M., Yatani A. (1992). Isolation and physiological characterization of taicatoxin, a complex toxin with specific effects on calcium channels. *Toxicon*.

[23] Nguyen T. T. N., Folch B., Létourneau M. (2014). Design of a truncated cardiotoxin-I analogue with potent insulinotropic activity. *Journal of Medicinal Chemistry*.

[24] Upadhyay S. K., Eckel-Mahan K. L., Mirbolooki M. R. (2013). Selective Kv1.3 channel blocker as therapeutic for obesity and insulin resistance. *Proceedings of the National Academy of Sciences of the United States of America*.

[25] Cheon S., Chung K., Lee K., An H. (2015). Bee venom suppresses the differentiation of preadipocytes and high fat diet-induced obesity through inhibiting adipogenesis. *Integrative Medicine Research*.

[26] Abbott M. J., Tang T., Sul H. S. (2010). The role of phospholipase A2-derived mediators in obesity. *Drug Discovery Today: Disease Mechanisms*.

[27] Tan N.-H., Lim K.-K., Nik Jaafar M. I. (1993). An investigation into the antigenic cross-reactivity of *Ophiophagus hannah* (king cobra) venom neurotoxin, phospholipase A_2_, hemorrhagin and l-amino acid oxidase using enzyme-linked immunosorbent assay. *Toxicon*.

[28] Huang M. Z., Gopalakrishnakone P., Kini R. M. (1997). Role of enzymatic activity in the antiplatelet effects of a phospholipase A2 from *Ophiophagus hannah* snake venom. *Life Sciences*.

[29] Harris J. B., Scott-Davey T. (2013). Secreted phospholipases A2 of snake venoms: effects on the peripheral neuromuscular system with comments on the role of phospholipases A2 in disorders of the CNS and their uses in industry. *Toxins*.

[30] Guilherme A., Virbasius J. V., Puri V., Czech M. P. (2008). Adipocyte dysfunctions linking obesity to insulin resistance and type 2 diabetes. *Nature Reviews Molecular Cell Biology*.

[31] Ntambi J. M., Kim Y.-C. (2000). Adipocyte differentiation and gene expression. *Journal of Nutrition*.

[32] Farmer S. R. (2006). Transcriptional control of adipocyte formation. *Cell Metabolism*.

[33] Nguyen T. T. N., Folch B., Létourneau M. (2012). Cardiotoxin-I: an unexpectedly potent insulinotropic agent. *ChemBioChem*.

[34] Park J.-E., Oh S.-H., Cha Y.-S. (2014). *Lactobacillus brevis* OPK-3 isolated from kimchi inhibits adipogenesis and exerts anti-inflammation in 3T3-L1 adipocyte. *Journal of the Science of Food and Agriculture*.

[35] Segula D. (2014). Complications of obesity in adults: a short review of the literature. *Malawi Medical Journal*.

[36] Marsh N., Williams V. (2005). Practical applications of snake venom toxins in haemostasis. *Toxicon*.

[37] Jain D., Kumar S. (2012). Snake venom: a potent anticancer agent. *Asian Pacific Journal of Cancer Prevention*.

[38] O'Shea M. (2011). *Venomous Snakes of the World*.

[39] Utkin Y. N. (2015). Animal venom studies: current benefits and future developments. *World Journal of Biological Chemistry*.

[40] Gregoire F. M., Smas C. M., Sul H. S. (1998). Understanding adipocyte differentiation. *Physiological Reviews*.

[41] Oh S. D., Kim M., Min B.-I. (2014). Effect of *Achyranthes bidentata* blume on 3T3-L1 adipogenesis and rats fed with a high-fat diet. *Evidence-Based Complementary and Alternative Medicine*.

[42] Hasani-Ranjbar S., Jouyandeh Z., Abdollahi M. (2013). A systematic review of anti-obesity medicinal plants—an update. *Journal of Diabetes and Metabolic Disorders*.

[43] Hwang D.-S., Kim S. K., Bae H. (2015). Therapeutic effects of bee venom on immunological and neurological diseases. *Toxins*.

[44] He Y., Li Y., Zhao T., Wang Y., Sun C. (2013). Ursolic acid inhibits adipogenesis in 3T3-L1 adipocytes through LKB1/AMPK pathway. *PLoS ONE*.

[45] Petras D., Heiss P., Süssmuth R. D., Calvete J. J. (2015). Venom proteomics of indonesian king cobra, *Ophiophagus hannah*: integrating top-down and bottom-up approaches. *Journal of Proteome Research*.

[46] Lei W., Zhang Y., Yu G. (2011). Cloning and sequence analysis of an *Ophiophagus hannah* cDNA encoding a precursor of two natriuretic pepide domains. *Toxicon*.

[47] Rajagopalan N., Pung Y. F., Zhu Y. Z., Wong P. T. H., Kumar P. P., Kini R. M. (2007). *β*-Cardiotoxin: a new three-finger toxin from *Ophiophagus hannah* (king cobra) venom with beta-blocker activity. *The FASEB Journal*.

